# Sex and gender-oriented colorectal cancer screening: a consensus study from the AIOM- GISEG working group

**DOI:** 10.3389/fonc.2026.1840003

**Published:** 2026-07-03

**Authors:** Marta Bianchini, Tiziana Vavalà, Francesca Rossi, Elsa Vitale, Manuel Zorzi, Stefania Gori, Rossana Berardi, Anna Maria Moretti, Marialuisa Appetecchia

**Affiliations:** 1 Oncological Endocrinology Unit, IRCCS Regina Elena National Cancer Institute, Rome, Italy; 2 AOU Città della Salute e della Scienza-Dipartimento di Oncologia, SC Oncologia 1U, Turin, Italy; 3 Department of Medical Oncology, Università Politecnica delle Marche, AOU delle Marche, Ancona, Italy; 4 Directorate of Health Professions and Nursing, ASL Bari, Bari, Italy; 5 Veneto Tumour Registry, Azienda Zero, Padua, Italy; 6 IRCCS Sacro Cuore Don Calabria Hospital, Negrar di Valpolicella, Verona, Italy; 7 Dipartimento di Pneumologia, Santa Maria Hospital, GVM Care & Research, Bari, Italy

**Keywords:** colorectal cancer, early detection, gender, gender oncology, screening, sex

## Abstract

**Introduction:**

Gender medicine in oncology focuses on understanding the differences in cancer incidence, progression and responses to oncological treatments between men and women. Colorectal cancer (CRC) is a paradigmatic example of sex and gender disparities in incidence and prevalence, age of onset, pathogenesis, tumour location and responses to oncological treatments. However, despite growing evidence of these differences, there are no current clinical recommendations based on sex and gender, nor are there differentiated diagnostic-therapeutic pathways.

**Methods:**

The objective of this consensus study was to identify areas of expert agreement regarding the potential appropriateness and clinical relevance of sex and gender-tailored colorectal cancer screening strategies on the basis of currently available evidence. A comprehensive literature review was conducted in order to examine any existing evidence on sex-and gender-related differences in CRC. Publications that were included in this review were manually searched in PubMed/Medline by using different combinations of relevant keywords. Based on synthesised evidence, a multidisciplinary panel of experts convened to develop consensus statements using the RAND/UCLA Appropriateness Method.

**Results:**

The findings highlighted significant disparities between men and women in incidence, age at onset, lesion distribution, participation rates, and barriers to screening, hence supporting the need for a more tailored approach in CRC screening strategies. Through a structured rating process and iterative discussion, the panel formulated a set of statements and actionable recommendations to enhance equity and effectiveness in CRC screening programs.

**Discussion:**

Core statements emphasize the importance of collecting and analysing sex-disaggregated data in screening registries, promoting further research to address existing knowledge gaps, tailoring communication strategies to gender-specific barriers and facilitators to improve screening adherence, considering potential differences between men and women in the optimal age and modality for CRC screening, within a framework of personalized and precision medicine.

## 1 Introduction

Gender medicine is defined by the World Health Organization (WHO) as the study of sex and gender differences that influence people’s health and disease. In fact, men and women express and experience many diseases due to biological (sex-related) and socioeconomic-cultural (gender-based) factors.

Women often face greater health challenges due to limited financial resources, lower employment levels, and greater caregiving responsibilities. For these reasons in clinical practice, a gender-sensitive approach should consider not only biological sex and age, but also ethnicity, cultural and socioeconomic conditions to provide further comprehensive care (1, 2).

Gender medicine in oncology focuses on understanding the differences in incidence, progression, outcome and toxicity of anticancer therapies between men and women.

Since the 1980s, studies started to exhibit that men and women had different incidence rates and prognoses for various types of cancer. Historically, lung cancer incidence was predominantly higher in men, but has been increasing in the last few decades in women, partially due to smoking habits. Moreover, biological differences, including different mutational profiles and responses to targeted therapies (e.g. against EGFR mutations), have been found (3–5).

Esophageal cancer incidence is higher in men than in women, given that biological differences in hormone levels, diet, and fat distribution may contribute to this disparity (6–8).

Even though bladder cancer is less common in women than in men, women are more likely to present worse outcomes (9).

Melanoma shows many sex and gender disparities regarding the anatomical localization (this disease is more frequent on the trunk in men and on the lower limbs in women) and prognosis (women generally have a better prognosis) stemming from biological and behavioural factors (10, 11).

There are significant explanations underlying sex differences in cancer. This sex disparity driven by a complex interplay between lifestyle and environmental factors, as well as biological factors, hormonal differences and genetic factors, such as the presence of two X chromosomes in females all influence cancer development and patient outcomes (12, 13).

Another reason is the difference in pharmacokinetics and pharmacodynamics. Males and females metabolize drugs differently due to variations in body composition, liver enzyme activity, and hormone levels. These differences affect the absorption, distribution, metabolism, and excretion of drugs, influencing their efficacy and safety. Females are more likely to experience toxicity from chemotherapy, requiring dose adjustments and supportive care strategies (14, 15).

Furthermore, estrogen and testosterone can significantly influence the growth and progression of certain cancers, such as breast and prostate cancer. Moreover, hormonal fluctuations can alter drug effectiveness and side effects (16).

Research also shows differences in immune responses to tumours, potentially affecting treatment outcomes (17, 18).

Following growing scientific evidence and awareness that men and women experience cancer differently, ESMO (European Society for Medical Oncology) organized the first multidisciplinary seminar on gender oncology in Switzerland in 2018. This first consensus on gender oncology was established through collaborative working groups among leading researchers, physicians and faculty members. It represented a landmark moment recognizing the critical differences in cancer between men and women and integrating sex and gender considerations into all aspects of oncological practice, encouraging the collection and analysis of sex-disaggregated data in oncological research (12).

Nowadays, both regulatory bodies, such as the Food and Drug Administration (FDA) and European Medicine Agency (EMA) and many scientific societies like ESMO or International Society for Gender Medicine (IGM) highlight the need for increasing women inclusion in clinical trials and emphasize sex as a biological variable in clinical research to ensure comprehensive safety and efficacy data.

Moreover, in the Italian context, scientific societies recognized the importance of gender and sex across all medical specialties. “GISEG”, the Italian Group of Health and Gender, is a Scientific Society that was founded in June 2009, with the aim of promoting the culture of Health and Gender Medicine, in accordance with the actions proposed by the Plan for the application and dissemination of Gender Medicine as provided by Article 3 of Italian law 3/2018.

“AIOM”, the Italian Association of Medical Oncology, is a professional organization dedicated to advancing the field of oncology in Italy. AIOM has been actively involved in integrating gender-specific considerations into cancer care, promoting research that explores how cancer affects men and women differently and developing clinical guidelines that incorporate gender-specific recommendations (19).

One of the main objectives of Gender Medicine and Gender Oncology is to integrate sex and gender into medical research and transfer these considerations into clinical practice, starting from planning of national screening programmes, because it is crucial for improving patient health.

Colorectal cancer (CRC) is a paradigmatic example of sex and gender disparity in tumour incidence and prevalence, age of onset, pathogenesis, primary tumour localization, and responses to oncological treatments (20).

However, despite the growing evidence of these differences, there are no different recommendations based on sex and gender, nor differentiated diagnostic-therapeutic pathways to date.

Sex and gender have a profound impact on CRC screening, affecting various aspects: risk factors for colorectal development and pathogenesis, participation rates in screening programs, the effectiveness of different screening methods.

Understanding these differences is crucial for developing more effective, personalized screening strategies, ultimately improving early detection and outcomes of CRC for both men and women.

For this purpose, a working group of health care professionals (experts in oncology and gender medicine, nominated by GISEG and AIOM scientific societies) has been established to collect and discuss scientific evidence in order to propose suggestions on how to better sex and gender-oriented screening.

## 2 Methods

A narrative literature review carried out to identify original studies on sex and gender differences in CRC, with particular focus on those aspects that may have a greater clinical and practical impact on the screening of this tumour.

The search conducted on PubMed/MEDLINE database, using the following queries:

(colorectal neoplasm [MeSH Terms]) AND ((“sex differenc*”) OR (“gender differenc*”));

colonoscopy AND ((“sex differenc*”) OR (“gender differenc*”));

sigmoidoscopy AND ((“sex differenc*”) OR (“gender differenc*”));

“fecal immunochemical test” AND ((“sex differenc*”) OR (“gender differenc*”));

“Fecal Occult Blood” AND ((“sex differenc*”) OR (“gender differenc*”)).

MesH terms detailed in Supplementary (**Supplementary Table 1**).

Furthermore, to explore also differences and inequities in ethnic minority and gender related health determinants, we conducted a separate bibliography search of scientific papers by querying on Pubmed/MEDLINE. This second search strategy is also available in **Supplementary Table 1**.

The following search filters were applied: Human studies; English language. The inclusion criteria for the selection of studies were: studies published before December 1, 2025; studies in English language; original studies on human aged over 18 years, including clinical trials, observational studies (retrospective and prospective), meta-analysis. Only studies reported sex and gender differences in CRC and screening were used for this article. We excluded articles referred to patients with non-oncological disease and studies not relevant for the topic of research (such as differences in treatment response).

Reference lists of included studies were also screened to identify any additional eligible articles, to describe the most robust scientific evidence on this topic.

A methodological support group reviewed evidence from literature, identified topics for the statements, collected and processed the data.

The objective of the consensus process was to identify areas of expert agreement regarding the potential appropriateness and clinical relevance of sex-tailored colorectal cancer screening strategies on the basis of currently available evidence and expert interpretation. Consensus was developed using the RAND/UCLA Appropriateness Method (University of California, Los Angeles) (21), a structured methodology that integrates the best available scientific evidence with expert judgment to assess the appropriateness of healthcare interventions, indicated in areas where high-quality randomized evidence may be limited, heterogeneous, or incomplete. A panel of six experts with recognized clinical and methodological expertise in the field of Gender Medicine, Oncology and Epidemiology, convened. Panel size was consistent with the minimum recommended number for application of the RAND/UCLA methodology. The panel was intentionally designed to include experts with specific competencies in Gender Medicine, directly aligned with the objectives of the present study.

A list of clinical indications (hereafter referred to as statements) related to CRC screening, was developed based on a narrative review of the literature. Clear and clinically specific statements were formulated to allow a consistent assessment of appropriateness across different clinical scenarios. The consensus process included two rating rounds.

Prior to the first round, all panelists received standardized written instructions regarding the interpretation of the 9-point Likert scale (1 = extremely inappropriate; 9 = extremely appropriate) and the criteria for rating agreement and appropriateness. In addition, a preliminary discussion was conducted to clarify the study objectives, statement structure, and scoring approach, in order to promote consistent interpretation across participants. In the first round, panelists independently and anonymously rated each statement using a 9-point Likert scale. A structured plenary meeting was subsequently held, during which aggregated first-round results were presented and areas of disagreement were discussed. In the second round, panelists re-rated the statements considering the group discussion. According to RAND/UCLA methodology, appropriateness was determined based on the median score and statements were classified as “appropriate” (median 7–9), “uncertain” (median 4–6 or any median with disagreement), or “inappropriate” (median 1–3). Consensus was considered achieved when statement ratings consistently fell within one of the three predefined appropriateness categories without disagreement. Disagreement was defined according to standard RAND/UCLA criteria as the presence of at least two ratings in the 1–3 range and at least two ratings in the 7–9 range.

## 3 Results

A total of 454 records were initially identified through the database search. 97 records were removed as duplicates. During the screening phase, 45 records were excluded based on title and additional 128 were excluded after abstract screening, 184 full-text articles were assessed for eligibility. Of these, 32 full-text articles were excluded (for not meeting inclusion criteria or lacking relevant outcome measures). Ultimately, 152 studies were included in the final analysis. In addition, 8 supplementary articles not identified through the PubMed search but considered relevant were manually included.

The 160 articles included in the analysis were divided into four topics:

- epidemiology and clinical characteristics (localization, age, stage and molecular features at diagnosis) of CRC;
- pathogenesis and risk factors;
- screening test performance;
- participation in screening programs.

For each of these topics, a series of statements were formulated. After the second round of ratings, for the 34 statements evaluated, 32 (94%) were classified as appropriate, and 2 (6%) as inappropriate. There were no disagreements observed.

### 3.1 Sex and gender differences in epidemiology and clinical characteristics

#### 3.1.1 Incidence

CRC is one of the most frequent causes of cancer-related deaths, with a higher incidence and mortality rate in men than women in different countries (22).

According to the latest estimates from the International Agency for Research on Cancer’s GLOBOCAN 2022 cancer statistics database, more than 1.9 million new cases of CRC worldwide (men 1,069,296 vs. women 856,822) and about 904.000 deaths (499,715 men and 404,144 women) were estimated to occur in 2022.

Considering only Europe, the incidence of CRC in 2022 was 538,584 new cases (289,251 men and 249,333 women) and the total number of deaths was 247,966 (133,609 men vs. 114,357 women) (23).

In regards to incidence, the proportion of male patients is greater than female ones across all age brackets and the ratio of male-to-female cancer cases has an increasing tendency with advancing age. However, the magnitude of the male predominance is age dependent. The rate ratios (RR) and rate differences (RD) do not remain constant over time: they increase gradually with age, peak at 70–74 years, and decline thereafter (24, 25).

Recent American data indicate that incidence and mortality rates are higher in men, with the male-to-female incidence rate ratio (IRR) varying by age: 1.1 (0–49 years), 1.4 (50–79 years), and 1.2 (≥80 years) (26).

Since 1990, the differences in incidence between genders have decreased in people aged 60 and older but have increased in younger age groups (40–59 years; p=0.003) (27).

#### 3.1.2 Localization

Several studies have demonstrated different localizations of primary tumours by sex, with males that exhibit higher rates of rectal and left-sided colon cancers, whereas females show a greater incidence of right-sided colon cancers (28, 29).

In a Dutch cohort of CRC patients diagnosed up to 2020, age-standardized incidence rates per 100,000 person-years were higher in males than females for both colon (41.2 vs. 32.4) and rectal cancer (22.8 vs. 12.6). Among colon cancer cases, right-sided tumours were more prevalent in females (59.2% vs. 48%), however, they more often had a normal BMI (body mass index) than males (30).

Similar data were seen in other countries and populations: among Korean patients, rectal cancer was more frequent in males (38.8% M vs. 31.8% F, p < 0.001), while right-sided colon cancer was more frequent in females (31.4% F vs. 22.7% M, p < 0.001). In this study, KRAS mutations were more common in females (34.4% vs. 23.3%) (31).

Moreover, tumour location varies across different age groups. Males aged 30–39, show a higher predisposition to proximal colon cancer compared to females. After 60 years, males exhibit an increasing proportion of rectal and distal colon tumours alongside a declining proportion of proximal colon tumours, trends not observed in females (32).

In the United States (over a 30-year period up to 2005), age-adjusted incidence of colon and rectal cancers declined from 60.5 to 46.4 per 100,000. The greatest reduction occurred in distal colon cancer (−37.8%), while proximal colon cancer showed minimal change (−6.4%) due to rising incidence in the ascending colon (24.8%) and hepatic flexure (21.3%). This increasing proportion of proximal CRC sites was more pronounced in females (33).

#### 3.1.3 Age

Regarding differences in age at onset, Chen et al. estimated overall and age-specific risk advancement periods (RAPs) for men compared to women, which quantify how many years earlier comparable levels of risk are reached by men. Men had a higher risk of CRC than women in the 50–69 age groups, while no significant differences were observed in the 40–49 age group, likely due to the strong influence of family history in this younger cohort (34).

Data from the largest cantonal cancer registry in Switzerland showed that women were older at diagnosis (median age at diagnosis of 73 years in women vs. 69 years in men). Therefore, in the age group ≥ 75 years more women were observed (55% versus 45%) (28).

Out of the 185.967 patients diagnosed with colon cancer in a study cohort in Germany, the women tended to be older across all tumour stages and had more poorly differentiated tumours (G3/4) than men and presenting a higher UICC stage (29). Authors suggested that women would need screening colonoscopies later than the usually recommended 75 years, as 27.3% of women in this cohort were older than 80 years when diagnosed with colon cancer.

In Italy, only individual monocentric or regional experiences exist, which stratify CRC cases by both sex and age. In a monocentric Italian study including 1603 CRC patients, women were older than men at the time of diagnosis with 56.6% of women older than 70 years, out of screening period, while the majority of men were detected during screening age (between 50 and 69 years in Italy). Moreover, this study confirmed that women showed a higher prevalence of right-sided cancer (37.6% F vs. 31% M; *p* < 0.05) (35).

Data from the 2022 Veneto Region Cancer Registry show that the proportion of people with cancer aged over 70 years is 62.3% among males and 63.9% among females (36).

Given that CRC incidence and mortality are higher in men than in women—who tend to reach comparable risk levels 4 to 8 years later (37), sex-specific initiation ages for screening colonoscopy have been proposed. The Asia-Pacific consensus guidelines recommend that women may begin screening at older ages due to the relatively low CRC incidence before 55 years (38).

#### 3.1.4 Stage and prognosis

Several epidemiological and clinical studies have demonstrated sex-specific differences in CRC prognosis and biological aggressiveness, with generally poorer outcomes observed in males. In particular, a large meta-analysis including over 1.3 million patients showed that females have significantly better overall survival and cancer-specific survival compared to males, suggesting a less aggressive tumour biology in females (39).

Recent population-based studies corroborate these findings, in which male sex was associated with higher mortality and reduced five-year survival, even after tumour stage, anatomical location, and treatment modalities adjustment (40).

Further recent analyses have identified male sex as an independent adverse prognostic factor, especially in early-onset of CRC, supporting the hypothesis of increased tumour aggressiveness in male patients (41). Overall, this body of evidence supports the concept that sex-dependent biological factors, including differences in hormonal exposure and estrogen receptor–mediated signalling, contribute to the observed disparities in tumour invasiveness, progression, and clinical outcomes in CRC.

Despite higher CRC incidence and mortality among men, some evidence suggests that women are at greater risk of late-stage diagnoses (42, 43). This is in part due to the women’s susceptibility to developing right-sided CRC and nonpolypoid that are detected less through regular screening and often asymptomatic at diagnosis (44).

A Canadian study explored the link between several individual- and environmental-level factors and the stage of CRC diagnosis. Among 267 patients affected by CRCs of a large prospective cohort study, 43.0% were diagnosed with stage III/IV. For men social support, having children, and caffeine intake were found to influence the stage; this suggests that lifestyle and social support were associated with CRC stage at diagnosis. For women a more complex set of predictors were identified, including family history of CRC, pregnancy, hysterectomy, menopausal hormone therapy and housework. These factors point to a variety of hormonal, reproductive, and lifestyle factors influencing the timing of CRC diagnosis in women (45).

Contrary to CRC, evidence regarding the differences in rectal cancer are scarce: only a Spanish study carried out on 770 patients undergoing surgery for rectal cancer reported a risk of having disseminated disease at diagnosis are higher among women (46).

#### 3.1.5 Molecular features

Both **s**ex and tumour location could influence molecular features such as PD-L1, MMR/MSI status and EGFR expression in CRC, suggesting a possible underlying mechanism of sex-specific colorectal carcinogenesis. In fact, in multivariate analysis PD-L1 expression was inversely correlated with males having proximal CRC, while females with proximal CRC showed a significant association with dMMR/MSI-high (OR 14.93, P = 0.032) and high EGFR expression (OR 4.17, P = 0.017) (47).

In a study published in 2023 that explored distinct molecular patterns of early-onset (age<50) non-hypermutated tumours (with fewer than 17.78 somatic mutations/1Mb) CRC by race/ethnicity and sex, authors studied a large cohort of 5,856 non-Hispanic White (NHW), 535 non-Hispanic Black (NHB) and 512 Asian/Pacific Islander. This study found significant differences in the mutation frequencies of LRP1B, FLT4, FBXW7, RNF43, ATRX, APC, and PIK3CA in early-onset non-hypermutated CRCs among different racial/ethnic groups. In addition, heterogeneity was observed for the effects of EP300, BRAF, WRN, KRAS, AXIN2, and SMAD2 regarding sex (48).

#### 3.1.6 Sex- and gender-based screening recommendations

Based on the aforementioned evidence regarding sex and gender differences in incidence, localization, age, stage and molecular features at diagnosis, the panel produced six statements, as reported in **Table 1**.

**Table 1:** Sex- and gender-oriented screening recommendations based on epidemiology and clinical characteristics .

Statement	Final classification	Median
Colorectal cancer screening is essential in both sexes to detect the tumor at an earlier stage and reduce mortality. To date there is a lack of recommendations on screening differentiated by sex and gender.	Appropriate	9
Disparities between men and women in the location, age of onset, and characteristics of colorectal cancer (CRC) should be given careful attention when designing and planning future preclinical and clinical studies.	Appropriate	9
Sex-related differences in CRC prevalence, tumor location, and natural history of the disease should be taken into account when selecting the primary screening protocols.	Appropriate	7.5
Earlier initiation (45–50 years) of colorectal cancer screening should be formally evaluated in a pre-planned prospective trial comparing men and women at average risk, given that men consistently show higher CRC incidence and mortality and reach equivalent risk levels several years earlier than women.	Appropriate	8
Extension of the upper age limit for colorectal cancer screening should be formally evaluated in pre-planned prospective studies among selected women in good general health, as women are diagnosed later than men, a substantial proportion receive a CRC diagnosis after 75years of age—often outside screening programs—and CRC prevalence is higher among women in age groups ≥75 years.	Appropriate	8
Different colorectal cancer screening intervals according to sex warrant evaluation in pre-planned prospective studies. Shorter intervals could be considered for male patients, given their higher incidence and greater biological aggressiveness and consequent greater benefit from more frequent screening, whereas standard intervals may remain appropriate for female patients. Stratification by additional factors, including age group and quantitative results of prior screening tests, should also be incorporated.	Appropriate	8

### 3.2 Sex and gender differences in pathogenesis and risk factors

The causes underlying the higher incidence of CRC in men remain poorly understood but may relate to greater exposure to risk factors such as physical inactivity, obesity, high intake of red meat and alcohol consumption, and smoking (49).

Men and women have different risk profiles: a cohort study investigating the risk factors identified some behavioural and metabolic features strongly associated with risk in men. Among these, alcohol consumption was significant higher in men with CRC than controls (50).

A pooled analysis of Japanese studies found smoking increases CRC risk at all subsites in men, while in women it is associated only with distal colon cancer (51).

In a French cohort of more than 200,000 adults, men have a significantly higher risk (OR 1.54) of developing CRC than women. A primary objective of the study is the attempt to understand how much of this difference can be attributed to social and behavioural factors rather than biology alone. About 30–50% of the observed differences in CRC risk between men and women may be at least partially explained by sex and gender-related factors: BMI was found to be one of the most important mediators, reflecting both biological and social aspects such as differences in dietary patterns and physical activity (52).

Obesity, linked to hyperinsulinemia and epithelial proliferation, is a recognized CRC risk factor, with evidence indicating sex-specific and anthropometric differences in tumour molecular pathways. Metabolic conditions like obesity, diabetes, and hypertension contribute to CRC risk, with variable effects between genders (53, 54).

As shown by a study about Korean adults, men with normal BMI and metabolic disorders have an elevated CRC risk compared to men without metabolic disease, even if their risk remained lower than that observed in obese individuals (55).

Obesity has been associated with an elevated risk of CRC, but the impact of long-term adiposity patterns and changes in body fat distribution remains insufficiently understood. Measures of central adiposity—such as increased waist circumference, hip circumference, and waist-to-hip ratio (WHR)—have been independently linked to a higher risk of CRC in men, even after BMI adjustment, while these associations are substantially attenuated or nullified in women. A 10-year increase in waist circumference is positively correlated with CRC risk only among men (p for trend 0.03 in men, 0.34 in women) (56).

In a large Korean nationwide cohort study, longitudinal changes in body weight were associated with the risk of colon cancer in a sex- and age-specific manner. Long-term weight gain of at least 5% was significantly associated with an increased risk of colon cancer in men, including younger individuals under 40 years of age when weight gain exceeded 20%, whereas substantial weight loss was associated with a reduced risk of colon cancer in women (57).

Moreover, waist circumference gain during adulthood may serve as an independent predictor of CRC risk in men, while in women excess body weight during early life may play a more critical role in CRC risk (58).

Visceral obesity is known to be involved in colorectal carcinogenesis. Silva et al. analysed how visceral adiposity could influence also staging and prognosis. High visceral adipose tissue is associated to smaller size, earlier stage colon cancers and lower nodal involvement only in males, without survival advantage. In females, there have been no associations found between visceral adipose tissue and tumour related characteristics (59).

Brandstedt et al. found a link between obesity-related anthropometric factors and the mutation status of KRAS and BRAF in primary CRC, stratifying data by sex. In males, several anthropometric measures were associated with both KRAS-mutated and KRAS wild type tumours and with BRAF wild type tumours. In females, only a high waist-hip ratio (WHR) increased the risk of KRAS-mutated CRC and only body fat percentage was found to be linked to BRAF wild type tumours (60).

Moreover, obesity is associated with MSS (microsatellite stability) CRC, showing some sex differences: MSS CRC significantly relates to high weight and BMI in females, and with high waist circumference in males (61). These results on the association between obesity and BRAF and microsatellite instability (MSI) status are consistent with previous studies (62).

Finally, a prospective study on 38,822 men and 37,357 women, shows that the influence of BMI on colon cancer risk differs between subsites in males (P = 0.02) but not in females (P = 0.95). In males, the hazard ratios (HRs) per 5 kg/m² increase in body mass index for proximal and distal colon cancer are 1.07 (95% CI: 0.86–1.33) and 1.49 (95% CI: 1.19–1.87), respectively. In females, the corresponding HRs are 1.15 (95% CI: 0.99–1.34) for proximal and 1.25 (95% CI: 1.05–1.49) for distal colon cancer. These findings highlight potential sex-specific differences in CRC risk, influenced by biological and environmental factors (63).

Another possible cause of the CRC differences observed in males and females is the sex-related variation in gut microbiota composition. Emerging evidence suggests that the intestinal microbiome, known to influence colorectal carcinogenesis, shows different profiles between male and female patients with CRC (64, 65).

As previously reported, literature suggested that colon carcinogenesis displayed sex differences in the incidence and site of tumours, as sex-steroids could have an essential impact on tumorigenesis of the large bowel. Estrogens and androgens exert regulatory effects on tissue homeostasis by influencing cellular growth, lineage-specific differentiation, and organ-specific functionality, notably within the gastrointestinal tract. Estrogen receptors (ERα and ERβ) influence cellular growth by regulating gene transcription in target cells and the two types (alfa and beta) receptors exhibit differential responses to estrogen (66, 67).

ERα is generally linked to proliferative and pro-tumorigenic signalling in several tissues, whereas ERβ counteracts ERα activity and exerts anti-proliferative and tumour-suppressive effects by inhibiting oncogenic transcription factors and promoting cell-cycle inhibitors. In the colon, ERβ is the predominant estrogen receptor, while ERα is minimally expressed and appears to have a limited role in CRC development (68, 69). ERβ expression is markedly reduced in CRC compared to normal mucosa in both sexes, showing a more pronounced decline in males (70–72). Loss of ERβ is associated with higher tumour grade, advanced stage, increased angiogenic signalling (e.g., VEGF-A), and poorer clinical outcomes, whereas ERβ-positive tumours are consistently linked to improved survival. More importantly, ERβ downregulation is already evident in precancerous lesions, suggesting a role in early tumorigenesis (73–82).

Sex hormones appear to confer a protective effect, as supported by evidence from hereditary nonpolyposis CRC, where males develop CRC at a significantly younger age than females. (38.8 vs. 47.2 years; p < 0.05) (83).

It is postulated that estrogens exert protective effects against the development of CRC through multiple mechanisms. A study shows that age-related DNA methylation of the estrogen receptor gene occurs in normal colon tissue and is present in all colorectal tumours, leading to gene silencing. This epigenetic change appears early in tumour development and may link aging to CRC by promoting neoplastic transformation (84). Moreover, experimental data further suggest that, at levels comparable to those observed in women undergoing hormone replacement therapy (HRT), estrogens can induce apoptosis in ERβ-expressing colon cancer cells, supporting a direct protective mechanism (85, 86).

Specific ERβ isoforms, particularly ERβ1, are associated with favourable tumour features including the MSI-high phenotype (87). During menopause, the decline in circulating estrogens may lead to upregulation of ERβ1, potentially contributing to the development of MSI-H tumours. Consistent with these findings, Breivik et al. reported age- and sex-related differences in the prevalence of MSI-H CRC, with older females exhibiting a higher frequency of MSI-H tumours compared to younger, an association not observed in males (88). Consistently, parity and hormone replacement therapy are associated with a reduced risk of MSI-high tumours (89).

Emerging and interesting evidence suggests sex-related differences in tumour invasiveness associated with the expression of melatonin receptors (MT1 and MT2) in CRC. Both receptors are known to mediate melatonin’s antiproliferative effects in several malignancies, such as breast cancer and CRC. The expression of melatonin receptors MT1 and MT2 is reduced in CRC tissues, particularly in males, and their loss is associated with increased tumour invasiveness (90–92). A reduction in melatonin receptor levels in male colorectal tumours, together with parallel changes in androgen receptor, ERα, and ERβ expression was observed, suggesting potential interaction between these pathways (93–95). Finally, sex differences have also been observed in KRAS-mutant CRC. KRAS mutations are more frequent in females overall (96), particularly around the menopausal transition, whereas in males they are more often associated with right-sided tumours and older age (97). Experimental evidence indicates a crosstalk between estrogen receptor signalling and the RAS pathway, suggesting that sex hormones may limit the expansion of KRAS-mutant clones. Declining hormone levels during menopause and andropause may therefore reduce this protective effect and contribute to age- and sex-specific CRC patterns (98, 99).

In conclusion, estrogen receptor signalling plays a pivotal, sex-dependent role in colorectal carcinogenesis and in the biological behaviour of tumours, accounting for many of the previously described differences in incidence, tumour location, and age at onset.

#### 3.2.1 Sex- and gender-based screening recommendations

Based on the aforementioned evidence regarding sex and gender differences in pathogenesis and risk factors, the panel voted on and approved seven statements, as reported in **Table 2**.

**Table 2:** Sex- and gender-oriented screening recommendations based on pathogenesis and risk factors.

Statement	Final classification	Median
Sex and gender should be systematically incorporated as a core variable in colorectal cancer risk stratification models, given the consistently higher incidence in men and the differential contribution of biological, metabolic and behavioral risk factors.	Appropriate	9
CRC prevention and screening strategies should account for gender-specific lifestyle and behavioral risk factors, particularly alcohol consumption, smoking, physical inactivity, and dietary patterns, which confer a disproportionately higher CRC risk in men.	Appropriate	8
Metabolic conditions—including obesity, diabetes, metabolic syndrome and visceral adiposity—should be explicitly considered in CRC risk assessment, with recognition of their stronger and more consistent association with CRC risk in men compared with women.	Appropriate	7.5
Emerging biological factors, including sex-related differences in gut microbiota composition, should be explored and progressively integrated into CRC risk models.	Appropriate	7
Long-term weight gain and active smoking, particularly in men and in younger individuals, should be considered a relevant risk modifier in colorectal cancer prevention strategies, potentially supporting tailored screening for high risk subjects.	Appropriate	8
The influence of sex steroids and estrogen receptor signaling on colorectal carcinogenesis should be acknowledged in CRC risk stratification and research frameworks, given their role in modulating tumor incidence, localization, and biological behavior.	Appropriate	7
A deeper understanding of sex and gender disparities in pathogenesis and in the effect of the risk factors it is necessary to improve prevention campaigns for CRC.	Appropriate	8.5

### 3.3 Screening performance of different tests according to sex

In many countries, screening of CRC consists of faecal tests for haemoglobin (FIT: faecal immunochemical test or gFOBT: guaiac faecal occult blood test), followed by colonoscopy in case of positive test results.

It has been demonstrated that many factors influence the values of faecal haemoglobin (fHb) in particular age and sex (100, 101), with higher concentrations in males than females in all age quintiles (102, 103). Possible explanations of this sex difference in median in faecal haemoglobin (fHb) concentration include lower blood Hb concentration levels in females; a more intense degradation of fHb in females caused by slower bowel transit time; and lastly more common use in men of drugs that stimulate bleeding (as aspirin and non-steroidal anti-inflammatory drugs).

Even if all sites and stages showed lower fHb values for females, these sex differences were significant only for left sided cancer and stage I cases (103).

Despite sex differences in median fHb, often the cut-off concentration used in screening programs correspond to the cut-off recommended by the manufacturer, without sex and age specific reference values. Only a few studies have evaluated the use of tailored and sex-specific fHb thresholds to maintain sex equality in screening performance indicators.

When setting positivity thresholds for quantitative FIT in screening programs, differences in fHb levels by sex, age, and region can have an impact on screening effectiveness. Lowering the threshold increases sensitivity for detecting neoplasia but also raises the false positive rate (104).

Sex-related differences in advanced CRC prevalence should be included when interpreting results and designing screening strategies, so sex-specific thresholds may improve detection accuracy and program efficiency accordingly (100, 105, 106).

In the Finnish screening program, individuals aged 60–69 were randomized into screening and control groups. Among men with left-sided tumours, screening was associated with lower lymph node and metastatic involvement, improved overall survival, and fewer non-radical resections and postoperative chemotherapy sessions compared to controls. These benefits were not observed in men with right-sided tumours or in women (107).

For the limits of fHb other screening techniques have been proposed and studied. Preliminary data suggest that the CD26 related serum biomarkers can be used in CRC screening context, but also for this kind of screening, it is important to consider sex differences because DDP4 activity is higher in males compared to females (108).

Other screening techniques consist of flexible sigmoidoscopy (FS). In a study population of 14,947 subjects, the sensitivity of FS in detecting CRC was higher in male patients (84% versus 70%) (109).

For men, primary screening by sigmoidoscopy (followed by colonoscopy in case of detection of distal adenomas), may be effective (110).

The 2023 study by Juul FE and colleagues evaluated the 15-year impact of FS screening on CRC. After long-term follow-up, screening was associated with a reduction in cancer incidence and mortality, with stronger protection for distal (left-sided) cancers than for proximal ones (111).

A greater incidence in proximal cancers in women could limit FS as a screening tool. In fact, in a large retrospective analysis, the authors found higher adenoma detection rate (ADR) observed in men (112).

Sex differences in quality indicators, such as cecal intubation rate (CIR) and adenoma detection rate (ADR), are also observed in colonoscopy.

Age-incidence of CRC is higher in men (21.1/1000000 versus 15.7/1000000), thus a higher number of malignant and pre-malignant lesions in men than women is expected in the screening program, and is one of the reasons behind a higher ADR in men (113–117). In Sarkeala et al. study, the detection rates per 1000 participants in a FIT-based screening program were 1.8 for CRC and 7.2 for advanced adenoma in male patients vs. 1.6 for CRC and 3.8 for advanced adenoma in female patients (118).

A greater number of adenomas occur in the right colon as well as a higher number of sessile serrated lesions in females, that are less likely to show blood in faecal tests and are less easy to detect during colonoscopy (119).

Moreover, other sex differences must be included. Colonoscopies can be technically more difficult in females due to a longer colon and more frequent previous pelvic surgery that could cause adhesions or angulations. In a cross-sectional retrospective analysis of 2,523 colonoscopy procedures, the adhesions responsible for obstruction were only in females (120). The cecal intubation rate is an index of successful and complete colonoscopy, and it is often lower in female patients (121).

In a cross-sectional analysis of 16,551 individuals, incomplete colonoscopies were less frequent in male than female patients, regardless of the endoscopist’s expertise (122).

The different rate of incomplete colonoscopies reported in literature is highly likely not because of bowel preparation quality. In fact, different studies showed that women had slightly better bowel preparation than men, with a higher proportion of adequate preparation, evaluated with the Boston Bowel Preparation Score (BBPS) (123–125). Those differences in cecal intubation time and completion rate, may instead be due to anatomical differences such as a longer transverse colon and smaller bowel diameter, which can make standard colonoscopes less suitable in females (126).

In clinical practice, the use of thinner colonoscopy devices in women improves tolerance and facilitates complete intubation to the ileocecal junction (127). Furthermore, a study demonstrated that using a small-caliber colonoscope reduces procedural pain in women and increases their willingness to undergo future unsedated colonoscopy (128).

A retrospective descriptive study conducted in Boston, on 16,573 screening colonoscopies, demonstrated that screening colonoscopy procedures required a longer time in women compared to men. (19.35 min vs 17.79 min, p <0.001) (123, 129).

Some studies have demonstrated higher sedation rates in women (130, 131). Another difference is that sedation increases CIR more in women (+2.95%) than in men (+1.28%); however, overall CIR remains lower in women (94.69% vs. 96.58%; P<0.0001). In summary, sedation improves CIR—particularly in women—but does not enhance ADR (p=0.12) (132).

In a multicentric study, 1,688 patients aged 40–49 and 5,090 patients aged 50–59 underwent colonoscopy over a 4-week period. Among individuals aged 40–49, the combined prevalence of adenomas and carcinomas was similar, while, in the 50–59 age group, male patients had a significantly higher prevalence (31.3% vs. 18.4%; P<0.0001) (133).

#### 3.3.1 Sex- and gender-based screening recommendations

Based on the aforementioned evidence regarding sex differences in the screening performance of various tests, the panel developed ten statements, as reported in **Table 3**.

**Table 3:** Sex- and gender-oriented screening recommendations based on screening test performance.

Statement	Final classification	Median
		
The use of appropriate screening tools should be optimized to reduce the risk of late diagnosis; for this purpose, it would be desirable to evaluate the applicability of different sex-specific approaches and protocols in CRC screening.	Appropriate	8
Future CRC screening research should be designed to prospectively evaluate sex-specific strategies, including tailored thresholds, modality choice, and initiation age, to improve effectiveness and equity.	Appropriate	8
CRC screening programs should incorporate sex and gender-stratified analyses in program evaluation, including participation, test performance, detection rates, complications, and outcomes.	Appropriate	8
Given the higher prevalence of right-sided and non-polypoid colorectal lesions—which carry a lower bleeding risk—in women, along with potential sex-related differences in colonic hemoglobin degradation, sex-specific fecal hemoglobin thresholds could be established. Further prospective studies are needed to determine and validate optimal sex-specific thresholds for fecal hemoglobin in screening programs.	Appropriate	7
Greater use of primary colonoscopy should be considered in women, as they have a higher incidence of right-sided, often non-polypoid tumors, limited detectability of proximal lesions by fecal tests and sigmoidoscopy.	Appropriate	8
Quality indicators for colonoscopy (e.g., cecal intubation rate, adenoma detection rate) should be monitored and reported separately by sex to identify and address systematic disparities.	Appropriate	9
In female patients, colonoscopy protocols should consider anatomical differences (e.g., longer colon, smaller diameter, prior pelvic surgery), including the use of thinner or more flexible colonoscopes to improve completion rates.	Appropriate	8
Female sex should be recognized as a factor affecting colonoscopy performance and duration, highlighting the need to account for longer procedural times and to implement tailored scheduling to ensure optimal colonoscopy efficiency.	Appropriate	8
Given the higher prevalence of right-sided adenomas and non-polypoid lesions in women, screening strategies should emphasize techniques and training that improve detection of non-polypoid and proximal lesions.	Appropriate	8
Sedation strategies should be optimized with awareness that sedation may improve cecal intubation rates more in women than in men.	Appropriate	7.5

### 3.4 Participation in colorectal cancer screening

It is well known that screening reduces disease incidence and mortality; however, current data on the comparative effectiveness of various screening strategies remain limited (134, 135).

Adherence to screening depends clearly on the type of test used. Regarding faecal occult blood test screening, published data are discordant: while most studies report no significant gender differences in participation, some indicate higher adherence among women, whereas others show greater participation among men (136–141).

The European Commission recently published EU quality assurance guidelines for cancer screening (including colorectal) and a follow-up report updating implementation data across European Member States (MSs) (142, 143).

A comparative analysis of CRC screening showed data from European Member States using fecal tests (FIT or gFOBT), where positive results led to subsequent colonoscopy.

The overall participation rate (considering all countries and all ages) was consistently higher among women than men for faecal occult blood tests. Data from 12 population screening programmes (6 using gFOBT and 6 using FIT) demonstrated that completion rates for endoscopic procedure were high—94.1% for FIT-based and 97% for gFOBT-based programs—but slightly lower in women (in women 92.3% and 96.8% respectively for FIT and gFOBT programs; in men 95.15% and 97.3%).

For screening programs where endoscopy was the initial test proposed, uptake was greater for FS than total colonoscopy (24.5% vs. 16.8%), with men being more likely to undergo endoscopic procedures (142, 143).

The Italian regional sigmoidoscopy program implemented a sequential strategy, offering FS once in a lifetime as the primary screening test, with biennial FIT provided as an alternative for individuals who declined FS (144). This type of combined approach significantly improved overall population coverage—from 24% to 40% in the reference year— without gender differences (135).

Several studies in literature, confirmed previous data that women are less likely to participate in endoscopic screening (137, 138, 145–147). A possible explanation for this gender difference could be related to gender-specific barriers to colonoscopy (148). Fear of perforation, embarrassment and bowel preparation are reported a*s* major barriers by women in different studies (149, 150). In addition, significantly more women than men fear a positive diagnosis, pain and risk concerns regarding colonoscopies (145, 151, 152). In contrast, unnecessary health care and uncomfortable vulnerability and physical invasiveness are mainly reported from men (149, 153, 154).

The higher use of FS by males (73% versus 67%) was partially explained by lower levels of socioeconomic deprivation, higher levels of marital status and lower perceived barriers in this type of screening (155, 156).

Among older Chinese adults, approximately 14% of men and 10% of women undergo FS or colonoscopy, with family history of cancer having a greater influence on screening participation in women (157).

Several studies have investigated through carrying out surveys the reasons for non-participation to screening programs. From these surveys, emerged factors influencing screening behaviour that are different in men and women (149, 158). Male non-participants frequently exhibit fatalistic attitudes and possess lower levels of knowledge and more misconceptions about cancer and fecal immunochemical test (FIT) screening. Female non-participants tend to hold negative perceptions and emotions regarding FIT screening, cancer, and healthcare providers. In addition, social factors adversely affect women’s decisions to undergo screening (159).

Many patients perceive colonoscopy procedures as stressful and painful and some studies showed gender differences in degree of anxiety levels experienced pre-procedure (160). A randomized controlled trial analysing the effects of sedative music on patients’ experience of anxiety, pain, relaxation, and well-being during colonoscopy, showed that sedative music reduced anxiety in women and increased well-being in men during colonoscopy (161).

Another source of gender differences in adherence may be a bias in reporting the type of screening performed. In fact, studies comparing self-reported screening data with medical records found that men are less reliable than women in accurately reporting sigmoidoscopy and colonoscopy, often overestimating their colonoscopy screening rates (162).

Participation and adherence are influenced by both patient- and physician-related factors. In a survey of 202 women aged 40–70, 43% preferred a female endoscopist for colonoscopy, while only 1.4% preferred a male physician and the remainder had no preference. Among those who preferred a female physician, 87% were willing to wait more than 30 days to obtain one, 14% were willing to pay extra, and 5% stated they would forgo colonoscopy altogether if a female endoscopist was not available. The authors concluded that preference for female physicians may delay colon cancer screening, representing a potential barrier to adherence (163).

Although research has not shown significant gender differences in medical practitioners’ recommendations for screening (164, 165), it is possible that the intensity of these recommendations varies. For example, in Germany, gynaecologists are the primary providers of fecal occult blood tests (FOBT) (93%) and patient education (61%) for women (166).

Knowledge of screening guidelines positively influences screening adherence in both men and women, although the factors associated with higher compliance vary by gender: older age in men, and a family history of cancer along with prior participation in other screenings (such as breast cancer screening) in women (167, 168).

In both gender, marital status was positively associated to screening participation compared to single or divorced people (141) while social isolation was negatively associated to screening (169).

Moreover in the European context, living alone and having lower social support is associated with lower screening uptake in both gender but it is more prominent in men and it is attenuated in countries with population -based screening programmes than countries in which screening is not systematically organized (170).

Different studies have demonstrated a correlation between screening uptake and socio-economic status (SES) (171, 172), geographic residence (173), ethnicity and racial minorities (174) and psychological factors (175, 176). We have reported below only studies that analysed these factors in relation to gender.

An American study examined the impact of increased health insurance coverage in men and women: a significant relative increase in lifetime colonoscopy use was observed only in women suggesting that barriers to CRC screening may differ by gender (177). A cross-sectional analysis of CRC screening uptake in England adults aged 60–69 years showed that uptake was correlated to the socio-economic status (SES), in women and older people. Moreover, there was lower uptake in the most ethnically diverse areas (38%) than other areas (52–58%), where these ethnic disparities were more evident in men (178).

The National Bowel Cancer Screening Program in South Australia highlighted inequities in participation based on gender, indigenous status, language spoken at home and geographical location (179).

In examining racial and ethnic minorities, it emerged that perceived medical discrimination is associated with lower screening rates for CRCs with gender differences: women perceiving medical discrimination were less likely to be screened for colorectal (OR = 0.66; CI = 0.64 - 0.69) differently from men. For this reason, it is important to identify gender-based and culturally specific strategies that may improve participation in cancer screening of patients from different backgrounds (180).

Moreover, findings show that there are differences in perceived risk for CRC between genders among African Americans, with women perceiving lower levels of risk than men (181).

Different authors suggested a better primary care distribution and healthcare system organization which could potentially reduce the ethnic/racial disparities in CRC screening (182–184).

#### 3.4.1 Sex- and gender-based screening recommendations

Based on the aforementioned evidence regarding gender differences in participation in CRC screening the panel produced eleven statements, as reported in **Table 4**.

**Table 4:** Sex- and gender-based screening recommendations based on participation in screening programs.

Statement	Final classification	Median
Participation, adherence, and completion rates for each screening modality should be systematically monitored and reported by sex and gender. Evaluation of gender-specific interventions designed to improve CRC screening uptake should be prospectively evaluated to determine their effectiveness and equity impact.	Appropriate	8
Despite the availability of screening programs, adherence remains suboptimal in both women and men, varies by screening modality, and is influenced by gender-specific barriers and facilitators, highlighting the need for gender-sensitive strategies to improve participation.	Appropriate	8.5
Public information and outreach efforts on sex and gender differences in colorectal cancer should be strengthened through gender-sensitive educational materials and invitation letters that address low knowledge and misconceptions more common in men and negative perceptions and emotional concerns more common in women.	Appropriate	8
Good medical advice and doctor-patient communication are important to improve acceptance and adherence to screening, however studies on the effectiveness of gender-specific promotion strategies are needed.	Appropriate	9
Socioeconomic factors and ethnicity/race play a key role in adherence to cancer screening programs. Policies and interventions addressing social inequalities may improve both acceptability and participation in CRC screening among disadvantaged and minority groups.	Appropriate	8.5
Given gender differences in uptake across colorectal cancer screening modalities, screening programs should implement gender-sensitive strategies to address male underparticipation in fecal test–based screening and female underparticipation in endoscopic screening.	Appropriate	8
The choice of the endoscopist’s gender, according to patient preference, in screening services could help increase colonoscopy adherence among women.	Inappropriate	3
For women, CRC screening promotion may benefit from integration with other preventive services, such as gynecological care or breast cancer screening programs.	Appropriate	8
Listening to relaxing music as non-pharmacological interventions should be considered during colonoscopy, to decrease anxiety among women and increase well-being among men.	Inappropriate	3
Screening programs should also be designed to facilitate access for people with low social support or social isolation.	Appropriate	7.5
Future research should prioritize comparative effectiveness studies of screening strategies stratified by sex, gender and social determinants, to inform evidence-based and equitable screening policies.	Appropriate	9

## 4 Discussion

Sex and gender differences significantly influence the epidemiology, clinical presentation, risk factors, and screening outcomes of CRC. Men have higher incidence and mortality rates than women and more frequently present with rectal and left-sided colon tumours.

Lifestyle factors differ by gender and can impact CRC risk differently; even hormonal differences may contribute to disparities in carcinogenesis and prognosis. Despite the importance of early detection, current screening guidelines are not tailored by sex or gender, and adherence remains low in both men and women, influenced by gender-specific barriers, socio-economic status, and ethnicity. In addition, common screening tests like sigmoidoscopy and fecal immunochemical tests are less sensitive in women, potentially due to anatomical differences and a higher prevalence of right-sided and sessile serrated lesions. These findings underscore the need for sex- and gender-sensitive approaches in CRC screening strategies to improve detection and outcomes.

Based on the available evidence in the literature the expert panel suggested directions for future preclinical and clinical research for the implementation of sex- and gender-based CRC screening and for the development of more effective and personalized screening strategies. Core statements emphasize the importance of collecting and analysing sex-disaggregated data in screening registries, promoting further research to address existing knowledge gaps, tailoring communication strategies to gender-specific barriers and facilitators to improve screening adherence, considering potential differences between men and women in the optimal age and modality for CRC screening, and monitoring the impact of gender-oriented interventions.

Some highly rated statements were supported predominantly by observational or registry-based studies rather than randomized controlled trials. However, this reflects the current state of evidence in the field of sex -tailored colorectal cancer screening, where long-term randomized data stratified by sex are still scarce. In this context, the appropriateness scores should not be interpreted as equivalent to guideline recommendation strength, but rather as an expert assessment of the balance between potential benefit, biological plausibility, and available evidence. The initial adoption of sex and gender tailored screening strategies may involve substantial organizational complexity and increased economic and operational burden, including modifications to laboratory workflows, adaptation of screening algorithms, and potential pressure on endoscopic capacity. These aspects may represent important challenges for established population-based screening programs, particularly in the short term. However, if validated, these approaches could provide significant long-term benefits, including improved risk stratification, more efficient allocation of healthcare resources, and enhanced screening effectiveness. Robust evidence from prospective studies designed for this issue, and health-economic evaluations are therefore needed to determine whether the anticipated clinical and public health advantages outweigh the initial implementation costs and organizational challenges, ultimately supporting their integration into future screening policies.

A notable finding from the currently available literature and international guidelines is the near absence of data regarding transgender and non-binary individuals. Gender-affirming hormonal therapies may plausibly influence colorectal carcinogenesis through estrogen- and androgen-mediated pathways, although evidence remains limited. Future research should specifically address CRC epidemiology, prevention, and screening strategies in transgender and non-binary individuals. This study has some limitations that should be acknowledged. First, the expert panel included a relatively small number of participants. However, the study was designed as a focused expert consensus initiative, and panel composition was intentionally restricted to experts with competencies considered most pertinent to the predefined research scope. Nevertheless, the absence of additional professional categories may have introduced selection bias and may limit the broader generalizability of the consensus findings. Furthermore, the use of a narrative rather than a systematic review, carries a risk of selection bias and incomplete retrieval of the available literature; however, given the exploratory nature of the present RAND UCLA consensus study, aimed at identifying educational needs and research priorities, a narrative approach was considered appropriate to provide a broad and flexible overview of the topic. In conclusion, this consensus provides a structured, evidence-informed framework to implement sex and gender oriented CRC screening strategies. The integration of sex- and gender-specific perspectives into CRC prevention and screening has the potential to improve the precision, appropriateness, and equity of healthcare interventions. Moreover, personalised and gender-aware strategies may also contribute to healthcare sustainability by improving adherence to screening and treatments, reducing inappropriate interventions and ultimately optimizing the use of healthcare resources, towards a personalized and precision medicine.

## Data Availability

The raw data supporting the conclusions of this article will be made available by the authors, without undue reservation.
