# Supplementary material for: Sex and gender-oriented colorectal cancer screening: a consensus study from the AIOM- GISEG working group
Source: Front Oncol. 2026 Jul 3;16:1840003. doi: 10.3389/fonc.2026.1840003 (PMC13375465; doi:10.3389/fonc.2026.1840003)
Supplement: Supplementary file 1 [file Table1.docx]

**Supplementary Material**

**Table S1:** **Search strategy**

| **Main search** |
| --- |
| **Search**  (colorectal neoplasm [MeSH Terms]) AND (("sex differenc*") OR ("gender differenc*"));  colonoscopy AND (("sex differenc*") OR ("gender differenc*"));  sigmoidoscopy AND (("sex differenc*") OR ("gender differenc*"));  "fecal immunochemical test" AND (("sex differenc*") OR ("gender differenc*"));  "Fecal Occult Blood" AND (("sex differenc*") OR ("gender differenc*")).  **Translations**  (colorectal neoplasm[MeSH Terms]): “Neoplasm, Colorectal” OR “Colorectal Tumors” OR “Colorectal Tumor” OR “Tumor, Colorectal” OR “Tumors, Colorectal” OR “Neoplasms, Colorectal” OR “Colorectal Cancer” OR “Cancer, Colorectal” OR “Cancers, Colorectal” OR “Colorectal Cancers” OR “Colorectal Carcinoma” OR “Carcinoma, Colorectal” OR “Carcinomas, Colorectal” OR “Colorectal Carcinomas”  (("sex differenc*"): “sex difference” OR “sex differences”  ("gender differenc*")): “gender difference” OR “gender differences” |
| **Secondary search** |
| **Search**  ("ethnic"[All Fields] OR "ethnical"[All Fields] OR "ethnically"[All Fields] OR "ethnicities"[All Fields] OR "ethnics"[All Fields] OR "ethnology"[MeSH Subheading] OR "ethnology"[All Fields] OR "ethnicity"[All Fields] OR "ethnology"[MeSH Terms] OR "ethnicity"[MeSH Terms]) AND ("colorectal neoplasms"[MeSH Terms] OR ("colorectal"[All Fields] AND "neoplasms"[All Fields]) OR "colorectal neoplasms"[All Fields] OR ("colorectal"[All Fields] AND "cancer"[All Fields]) OR "colorectal cancer"[All Fields]) AND ("gender identity"[MeSH Terms] OR ("gender"[All Fields] AND "identity"[All Fields]) OR "gender identity"[All Fields] OR "gendered"[All Fields] OR "gender s"[All Fields] OR "gendering"[All Fields] OR "genderized"[All Fields] OR "genders"[All Fields] OR "sex"[MeSH Terms] OR "sex"[All Fields] OR "gender"[All Fields]) AND ("sex characteristics"[MeSH Terms] OR ("sex"[All Fields] AND "characteristics"[All Fields]) OR "sex characteristics"[All Fields] OR ("sex"[All Fields] AND "differences"[All Fields]) OR "sex differences"[All Fields]) AND ("disparate"[All Fields] OR "disparately"[All Fields] OR "disparities"[All Fields] OR "disparity"[All Fields]) AND ("inequalities"[All Fields] OR "inequality"[All Fields] OR "inequities"[All Fields] OR "inequity"[All Fields]) AND ("inequalities"[All Fields] OR "inequality"[All Fields] OR "inequities"[All Fields] OR "inequity"[All Fields]) AND ("disparate"[All Fields] OR "disparately"[All Fields] OR "disparities"[All Fields] OR "disparity"[All Fields]) |
